# Identification of recurrence marker associated with immune infiltration in prostate cancer with radical resection and build prognostic nomogram

**DOI:** 10.1186/s12885-019-6391-9

**Published:** 2019-12-03

**Authors:** Xin Rui, Siliang Shao, Li Wang, Jiangyong Leng

**Affiliations:** Department of Urology, HwaMei hospital, University of Chinese Academy of Sciences, (Ningbo No. 2 Hospital), 41 Xibei Street, Ningbo, 315010 Zhejiang Province China

**Keywords:** Prostate cancer, Immune infiltration, Nomogram, Th2 cells, Tcm cells

## Abstract

**Background:**

Some historic breakthroughs have been made in immunotherapy of advanced cancer. However, there is still little research on immunotherapy in prostate cancer. We explored the relationship between immune cell infiltration and prostate cancer recurrence and tried to provide new ideas for the treatment of prostate cancer.

**Methods:**

Prostate cancer RNA-seq data and clinical information were downloaded from the TCGA database and GEO database. The infiltration of 24 immune cells in tissues was quantified by ssGSEA. Univariate Cox regression analysis was used to screen for immune cell types associated with tumor recurrence, weighted gene co-expression network analysis (WGCNA) and LASSO were used to identify hub genes which regulate prognosis in patients through immune infiltration. Then, the nomogram was constructed based on the hub gene to predict the recurrence of prostate cancer, and the decision curve analysis (DCA) was used to compare the accuracy with the PSA and Gleason prediction models.

**Result:**

Analysis showed that Th2 cells and Tcm related to prostate cancer recurrence after radical prostatectomy, and they are independent protective factors for recurrence. Through WGCNA and Lasso, we identified that NDUFA13, UQCR11, and USP34 involved in the infiltration of Th2 cells and Tcm in tumor tissues, and the expression of genes is related to the recurrence of patients. Based on the above findings, we constructed a clinical prediction model and mapped a nomogram, which has better sensitivity and specificity for prostate cancer recurrence prediction, and performed better in comparison with PSA and Gleason’s predictions.

**Conclusion:**

The immune cells Th2 cells and Tcm are associated with recurrence of PCa. Moreover, the genes NDUFA13, UQCR11, and USP34 may affect the recurrence of PCa by affecting the infiltration of Th2 cells and Tcm. Moreover, nomogram can make prediction effectively.

## Background

Prostate cancer (PCa) is the most common cancer among men in developed countries and the eighth leading cause of cancer death in the world [1]. PCa is a malignant tumor of the male reproductive system. It ranks second among male fatal malignancies in Western countries, second only to gastric cancer [2]. In China, with the aging of the population and the improvement of diagnostic techniques, the incidence of prostate cancer has increased significantly in recent years, and it has the trend of rejuvenation gradually. The cause of prostate cancer is still unclear, and its mechanism of development is a complex process involving multiple factors. At present, the treatment of prostate cancer includes radical surgery, external beam radiotherapy, brachytherapy, experimental prostate cancer topical treatment, endocrine therapy, and chemotherapy, etc. [3–8]. As surgical techniques mature, the incidence of complications and patient mortality have been declining. However, approximately 40% of patients still relapse within 5 years after surgery, and most are young patients who expect longevity. About 27 to 53% of patients eventually develop local recurrence or distant metastasis within 10 years after surgery [9–11].

In the past decade, research on immunotherapy for advanced cancer has made some historic breakthroughs, especially in Melanoma, Non-Small Cell Lung Cancer, etc. [12, 13]. In Hussein’s [14] study, it was pointed out that a variety of different immune cells were detected from prostate cancer tissues, including natural killer cells, CD4+ and CD8+ T-cells, dendritic cells and tumor-associated macrophages. At the same time, Kiessling’s [15] research indicates that the prostate also harbors multiple tumor-specific antigens, such as PSA, prostate acid phosphatase (PAP), and prostate-specific membrane antigen (PSMA), among others. These have provided the motivation and goals for further research on prostate cancer immunotherapy.

This study intends to quantify the degree of immune cell infiltration in prostate cancer tissues, and use univariate COX regression analysis to analyse the relationship between immune cells and tumor recurrence. The weighted gene co-expression network analysis (WGCNA) was used to identify genes associated with type 2 T helper (Th2) cells and central memory T cell (Tcm) in immune cells. The Least absolute shrinkage and selection operator (LASSO) method was used to screen out key genes about tumor recurrence and decision curve analysis (DCA) was used to compare the predictive model accuracy of model of two immune infiltrating cells and three key genes with PSA and Gleason. Finally we construct a nomogram for predicting prostate cancer recurrence.

## Methods

### Data collection

This study is intended to describe the relationship between recurrence and immune infiltration after radical resection of prostate cancer. Therefore, we downloaded the raw gene expression profile and clinical data of Prostate Adenocarcinoma (TCGA-PRAD) from The Cancer Genome Atlas (TCGA). A total of 266 samples were selected. The inclusion criteria for the selected samples were: samples with negative surgical margins for the cancer (Histological type is Prostate Adenocarcinoma Acinar Type, and the residual tumor is R0 (R0 is negative surgical margins for cancer)). Raw gene expression profile and clinical data were obtained. FPKM (Fragments Per Kilobase Million) of the GSE54460 data set was downloaded from the Gene Expression Omnibus (GEO) database (HTTP:// www.ncbi.nlm.nih.gov/geo/): a total of 106 samples, of which 61 were R0 samples. In the next study, the data extracted from the TCGA database was used as the experimental group, and the GEO database data was set as the verification group, and the two sets of data were analyzed separately.

### Infiltration of immune cells

The TCGA and GEO gene expression profile data were used to quantify the infiltration of immune cells in tumor tissues by ssGSEA (Single-sample gene set enrichment analysis), and the infiltration of 24 immune cells was obtained. ssGSEA, which computes an Enrichment score representing the degree to which genes in a particular gene set are coordinately up- or down-regulated within a single sample. The ssGSEA ranks the genes by their absolute expression in a sample and computes Enrichment score by integrating the differences between the empirical cumulative distribution functions of the gene ranks [16, 17].

### Univariate cox regression analysis and Kaplan -Meire’s curve

We used the “survival” package of R to do the Cox single factor analysis and selected the immune cells which may affect the recurrence of prostate cancer in both TCGA and GEO datasets (*p* < 0.05). Next, the “survminer” package was used to perform the best separation statistic, which divides genes expression into high and low groups according to best separation, then make the Kaplan-Meier curve.

### Weighted gene co-expression network analysis (WGCNA)

First, screen out the highest 1/4 of the gene expression of variance by the quartile of gene expression level, that is, the highest 1/4 gene with a significant difference in gene expression in tissues. Then, the expression profile and clinical information of the above-mentioned integrated genes are used as input data sets of WGCNA, and a sample clustering tree map is constructed, and two outlier samples are eliminated, and a sample tree diagram and a trait heat map are constructed to express similar genes. The spectrum is divided into different gene modules. Then, the gene expression profile and clinical information of the above-mentioned integrated genes were used as input data sets of WGCNA, and construct the sample dendrogram and trait heatmap after two outlier samples were excluded, and the similar gene expression profiles were divided into different gene modules. When using the pick Soft Threshold function to calculate β from 1 to 20, the logarithm of the node connection log(k) and the logarithm of the probability of k is log(p(k)) R^2^. Ensure that the average connectivity of the network (mean.k) is above a certain level, and the corresponding β of R^2^ is the best soft threshold. The network at this time can obey the scale-free criteria without affecting the network connectivity. Considering this, this study selects β = 12 as the soft-thresholding.

After determining the soft-thresholding, we build the network. The adjacency matrix is transformed into a topological overlapping matrix to construct a network, and the gene dendrogram and nodule color are established by using the degree of dissimilarity. To further analyze the module, we calculated the dissimilarity of the module eigengenes, hierarchically clustered the modules, and merged similar modules. Set the minimum number of genes in the module to 50. Divide the initial module by dynamic tree shearing, set abline = 0.25, merge the modules with the high similarity of feature genes in the gene dendrogram, and finally get 11 modules.

We included age, survival time, survival status, time of recurrence, and recurrence status in clinical information as relevant variables. The Pearson correlation coefficient between the sample vector of these variables and the characteristic gene of the module is calculated to measure the degree of association between the clinical features and the module, and to make a correlation analysis diagram between the gene module and the clinical information. Find out which modules of immune cell genes are most relevant to tumor recurrence. Moreover, calculate the connectivity within the module, select the 30 genes with the highest connectivity for further analysis.

### Screening key genes by LASSO and survival (relapse) analysis

Next, we used the Lasso of “Glmnet” package to further screen the 30 related genes. Survival (relapse) analysis of key genes was performed using “survival” and “survminer” packages to verify whether differently expressed genes differed from tumor recurrence.

### Decision curve analysis for comparingthe prediction effects

Decision curve analysis was used to compare the predictive model accuracy of model of two immune infiltrating cells and three key genes with PSA and Gleason. Decision curve analysis graphically displays the clinical utility of each model, based primarily on the potential threshold for recurrence risk (x-axis) and the net benefit (y-axis) using the model.

### The establishment and evaluation of the nomogram

Integrate the factors associated with tumor recurrence and create the nomogram of TCGA and GEO data by R. The returned samples are taken by Bootstrap self-extraction, and then the sampled samples are verified by calculation. Calculate the C index of the prediction model separately. The predictive power of the model was assessed and quantified by measuring the extent to which the C-index and the baseline time predicted by the nomogram in the standard curve fit the actual recurrence time. Finally, made the ROC curve of prostate cancer recurrence, and evaluated the accuracy of the nomogram.

## Results

### Quantify immune cell infiltration and analyze the relationship between immune cells and tumor recurrence

**Fig. 1:**
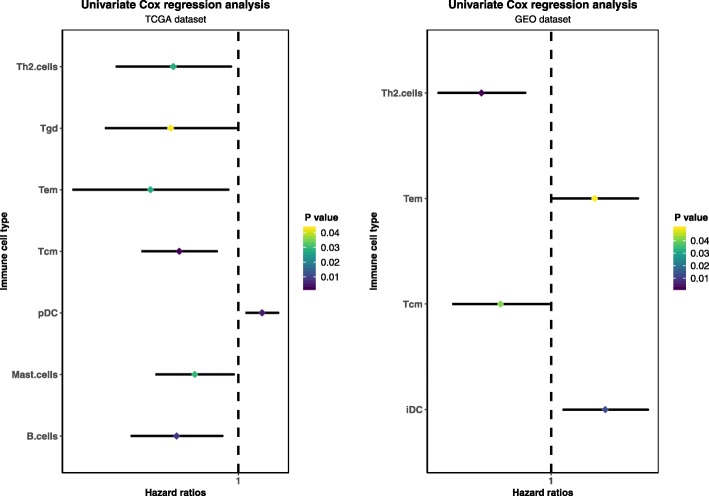
Univariate COX regression analysis of immune cells cox in TCGA and GEO data, Screening data *p* < 0.05. The immune cells Th2 cells and Tcm had the same results in TCGA and GEO data analysis, all of which were HR < 1, indicating that the two immune cells play a protective role in prostate cancer recurrence

We used ssGSEA to quantify mRNA data for immune cell infiltration. Finally, 24 infiltrating immune cells were obtained including: aDC, B cells, CD8 T cells, Cytotoxic cells, DC, Eosinophils, iDC, Macrophages, Mast cells, Neutrophils, NK CD56 bright cells, NK CD56dim cells, NK cells, pDC, T Cells, T helper cells, Tcm, Tem, TFH, TFH, Tgd, Th1 cells, Th17 cells, Th2 cells, Treg. Univariate Cox regression analysis was used to identify immune cells associated with recurrence after radical resection of prostate cancer, the results are shown below (Fig. 1). In order to find out the relationship between immune cell infiltration and prostate cancer recurrence, Hazard ratios > 1 is a risk factor for recurrence, and Hazard ratios < 1 is a recurrence protection factor. The type of immune cells screened must be consistent with the Hazard ratios performance in the experimental and validation groups. The immune cells Th2 cells and Tcm cells had the same results in TCGA and GEO data analysis, all of which were HR < 1, indicating that the two immune cells play a protective role in prostate cancer recurrence. The immune cell Tem was an independent protective factor in the TCGA group and the independent risk factors in the GEO group. Considering the contradiction between the two, the Tem was not included in the follow-up study.

**Fig. 2:**
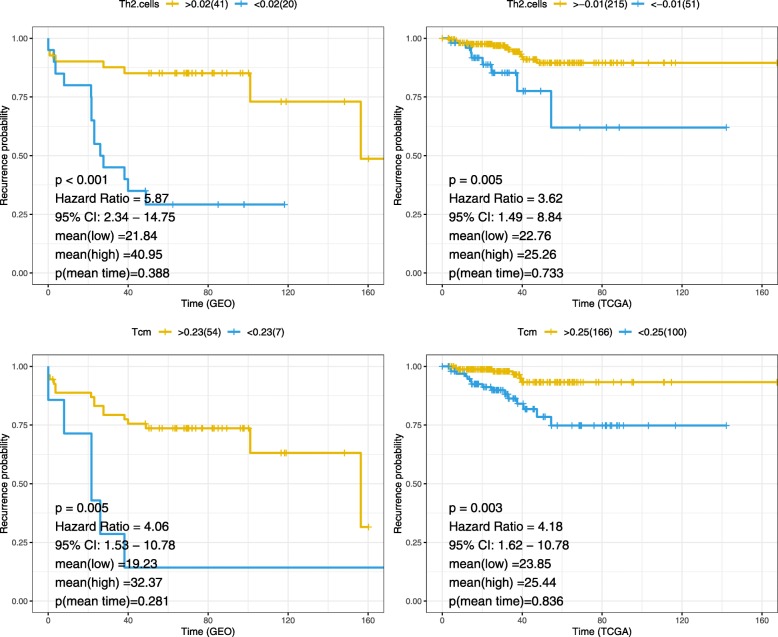
Kaplan-Meier curves of Th2 cells and Tcm in immune cells. Genetic analysis in the TCGA and GEO databases showed that the high infiltration group had a significant inhibitory effect on prostate cancer recurrence, while the low infiltration group had the opposite effect(*p* < 0.05). Mean (low) and mean (high) represent the mean time to recurrence of low and high immune cells infiltration group

Using the “survival” and “survminer” package to determine the relationship between the two immune cells and prostate cancer recurrence, and make the Kaplan-Meier curve, found that the higher the degree of Th2 cells and Tcm infiltration, the lower the recurrence rate of prostate cancer. Mean time to recurrence shows that the patient in high infiltration group are earlier recurrence, but the time to recurrence of low and high infiltration shows no significant difference. The results are shown in the following Fig. 2.

### The weighted gene co-expression network analysis (WGCNA) construction and key module identification

**Fig. 3:**
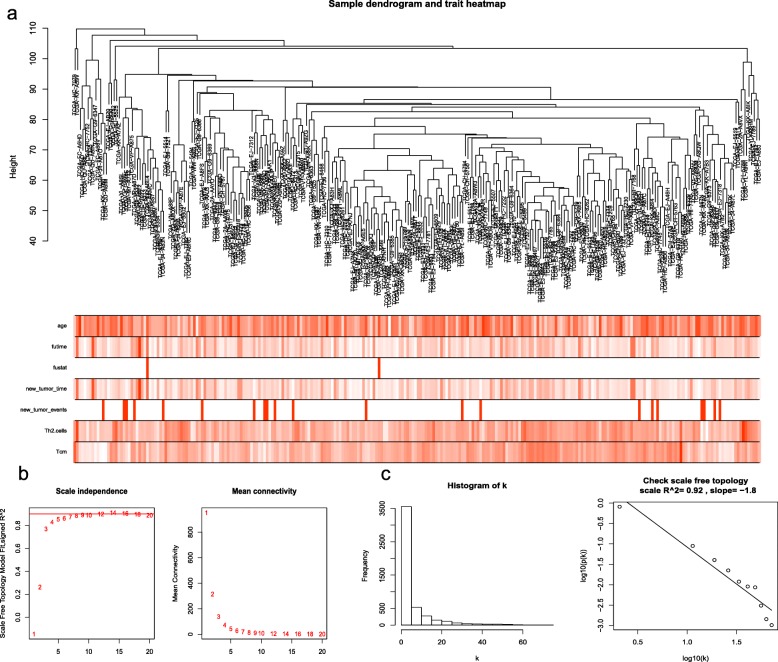
**a** Sample tree and trait heat map with Th2 cells and Tcm as two variant samples. The five clinical information included were age, survival time, survival status, recurrence time, and recurrence status. The larger the value in the above figure, the darker the color. White is no recurrence; red is recurrence. **b** and **c** Determination of soft-thresholding power in the weighted gene co-expression network analysis (WGCNA)

The highest 1/4 of the gene expression of variance was screened by the quartile of gene expression level, and 4855 genes were screened out from 19,418 genes. Through the above steps, the expression profile and clinical information of the gene are integrated as the input data set of WGCNA, and two outlier samples are eliminated according to the sample clustering tree, and the sample dendrogram and trait heatmap are constructed (Fig. 3a).

After discarding two outlier samples, WGCNA was performed on the 4855 most variable genes. Soft threshold power was set to 12, in which R^2^ was 0.92, ensured a scale-free network (Fig. 3b, c).

**Fig. 4:**
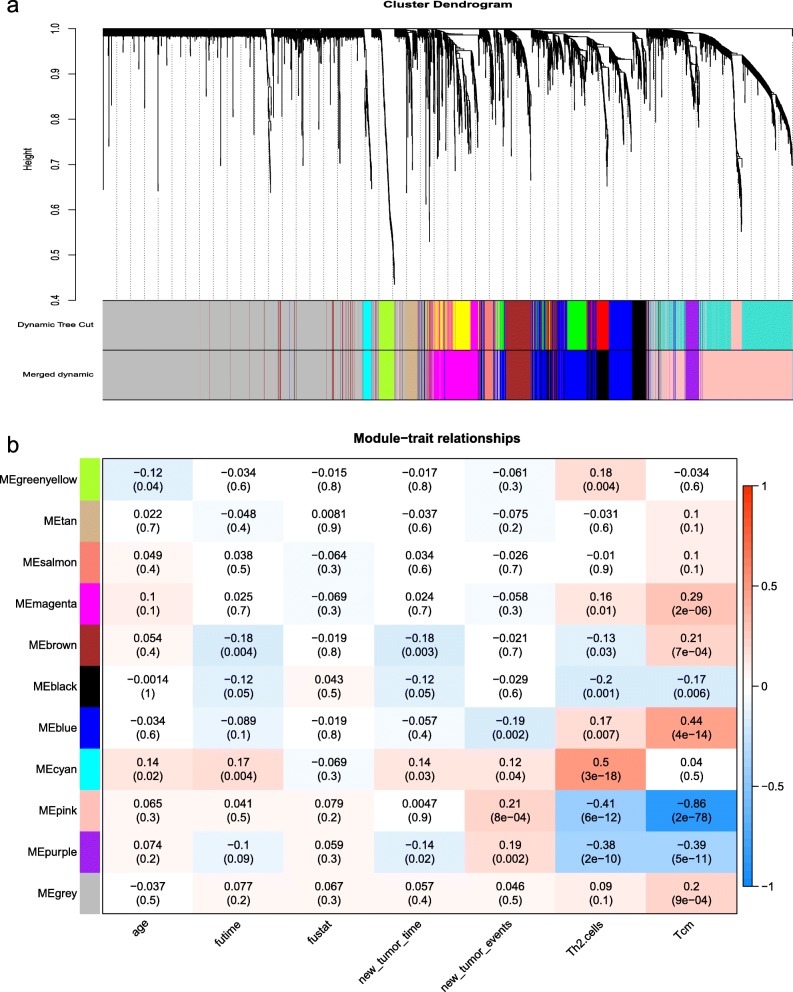
**a** The weighted gene co-expression network analysis (WGCNA):the gene dendrogram and nodule color. **b** Correlation between gene modules and clinical information

After determining the soft-thresholding, we build the network. Set the minimum number of genes in the module to 50. Divide the initial module by dynamic tree shearing, set abline = 0.25, merge the modules with the high similarity of feature genes in the gene cluster dendrogram, and finally get 11 modules (Fig. 4a). Each color represents a different module, where gray represents the genes that cannot be assigned to the module, the upper layer is the initial module obtained by the dynamic tree shearing method, and the lower layer is the combined final module.

Analyze the correlation between clinical information and key genes in the module and create a heat map (Fig. 4b). We found that the correlation coefficient between the pink module and Tcm was the highest (Pearson Cor = − 0.86, medium intensity negative correlation, and significant correlation). This indicates that the genes in this module are most relevant to tumor recurrence. The absolute values of the correlation coefficient between genes and age, survival time, survival status, recurrence time and recurrence status in other modules were small, indicating that the relationship between genes and clinical information was weakly correlated or irrelevant. Next, we calculated the connectivity within the pink module and selected the 30 most connected genes for further analysis.

### Screening the key genes and survival analysis

**Fig. 5:**
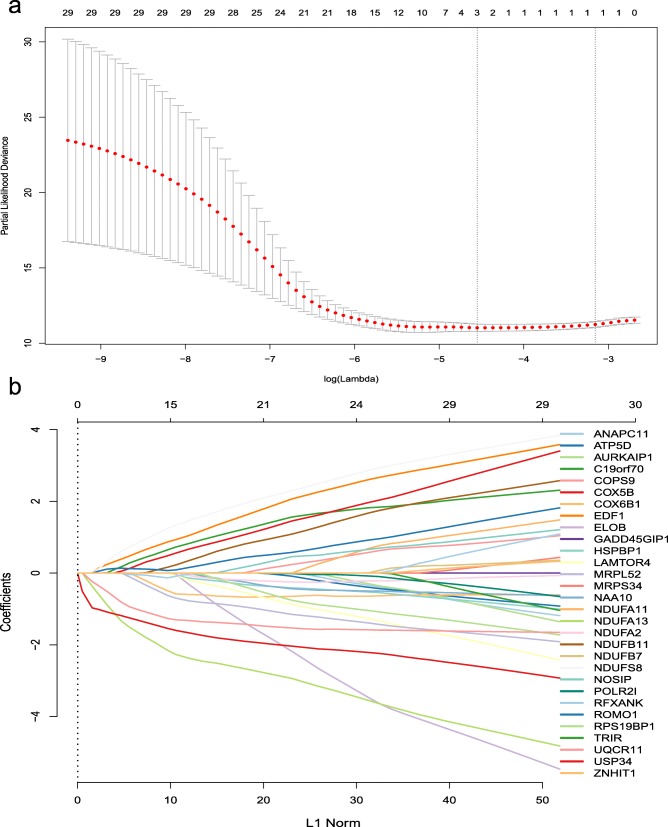
**a** Partial likelihood bias of the LASSO coefficient distribution. The vertical dashed line indicates the minimum partial likelihood deviation. **b** Distribution of LASSO coefficients for 30 related genes

**Fig. 6:**
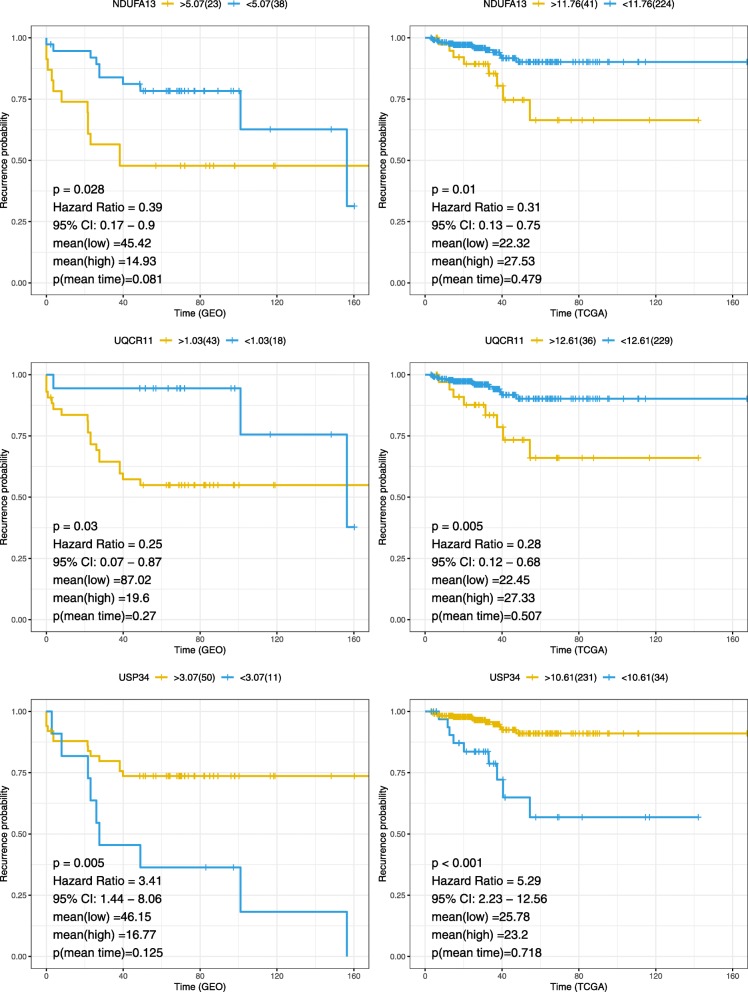
Kaplan meiers of the genes NDUFA13, UQCR11, and USP34 in TCGA and GEO datasets. Mean (low) and mean (high) represent the mean time to recurrence of low and high gene expression

Then we use LASSO to perform the next screening of the above 30 genes (Fig. 5). Using the cross-validation method to estimate the estimated adjustment parameter λ, when λ = 0.004570069, the error rate is the minimum. At this time, selected three key genes: NDUFA13, UQCR11, and USP34. Then, the survival (relapse) analysis of the three key genes was carried out separately (Fig. 6). As a result, the high expression of the USP34 has a protective effect on prostate cancer recurrence in TCGA and GEO gene expression profile, and the gene NDUFA13, UQCR11 is the opposite. Mean time to recurrence shows different in low and high expression group, and the time to recurrence of low and high gene expression shows no significant difference.

### Comparison of prediction model accuracy

**Fig. 7:**
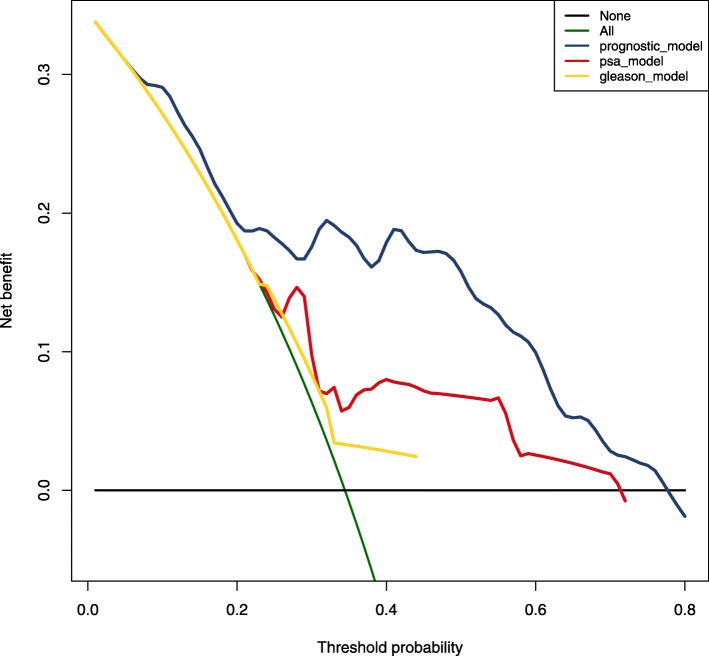
Decision curve analysis for the model of two immune infiltrating cells and three key genes, PSA-model, and Gleason risk prediction models

Finally, decision curve analysiswas used to compare the prediction model accuracy between different predictive models. As shown in Fig. 7, the decision curve analysis can graphically display the clinical utility of each model (where the x-axis represents the risk of recurrence and the y-axis represents the net benefit. In this analysis, compared to the PSA and Gleason scores, model of two immune infiltrating cells and three key genes shows better prediction accuracy .

### Establishment and evaluation of clinical predictive models

**Fig. 8:**
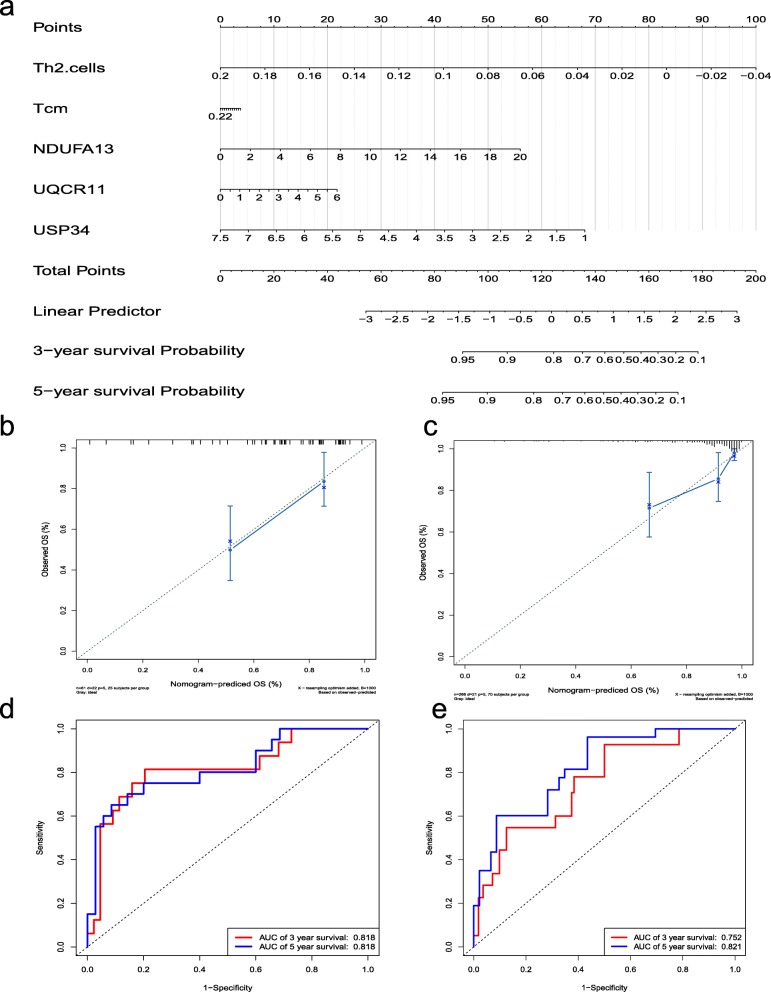
**a** Nomogram of postoperative recurrence of prostate cancer. The five predictors include two immune cells, Th2 cells, Tcm, and three key genes, NDUFA13, UQCR11, and USP34. Each factor corresponds to its own score, and each score is added to obtain a total score. The 3-year and 5-year recurrence rates of the total scores indicate the 3-year and 5-year recurrence rates of the patients. **b** and **c** Calibration curve for 5-year recurrence rate of prostate cancer in GEO and TCGA datasets. **d** and **e** ROC curves for 3 year and 5 year recurrence in GEO and TCGA datasets

Combine the above two immune infiltrating cells and three key genes to construct a nomogram. Each factor in this nomogram is given a certain score. According to the actual situation of each sample, the score corresponding to each prognostic factor is added to obtain a total score, and the total score corresponds to the corresponding scale. It is possible to obtain a 3-year and 5-year recurrence rate of the patient (Fig. 8a).

The C-index index of the calculated prediction model is TCGA (0.825) and GEO (0.757), respectively. The predictive p ower of the nomogram model is evaluated and quantified by measuring the degree of fit between the C-index and the baseline time predicted by the nomogram in the standard curve. As can be seen from the 5-year recurrence calibration curve shown in Fig. 8b, c, the nomogram model has a better predictive effect on recurrence.

At the same time, based on five relevant factors, the risk scores of the patient’s postoperative recurrence were calculated and the receiver operating characteristic (ROC) curve was plotted (Fig. 8d, e). The results showed that the TCGA data had a recurrence of 3 years and 5 years, and the area under the curve was 0.752 and 0.821, respectively. The GEO data showed recurrence at 3 and 5 years, and the area under the curve was 0.818 and 0. 818. The larger the area under the curve, the better the prediction effect.

## Discussion

In recent years, the incidence of prostate cancer has become higher and higher, and it has become the most common solid cancer in men. Its diagnostic rate is about 12%, and it accounts for 9% of male cancer deaths [18]. In countries where prostate-specific antigen (Psa) screening is widespread, postoperative biochemical recurrence rates for prostate cancer are approximately 1/3. In countries where PSA screening is not widely adopted, the incidence of diagnosis of metastatic prostate cancer is significantly higher than in other countries, and the metastatic prostate cancer eventual emergence of castration resistance results in the lethal phenotype termed metastatic castration-resistant prostate cancer (mCRPC). These patients have a median overall survival (OS) between 12 and 36 months [19]. The treatment prospects of prostate cancer are not satisfactory, and different treatment strategies are needed to improve the survival rate of patients with advanced prostate cancer.

As we have seen before, a variety of immune cells and tumor-specific antigens have been detected in prostate cancer tissues. These provide the goal of immunotherapy for prostate cancer. Studies have shown that after treatment of metastatic prostate cancer with relevant immunologically active substances, the overall survival rate of patients is significantly improved compared with the control group [20, 21]. This has also led more and more scientists to focus on the study of prostate cancer and immune mechanisms. The current research results show that reasonable vaccine treatment is essential for optimizing the efficacy of prostate cancer. Moreover, clinical data from multiple tumor types suggest that cancer vaccines work best in the early stages of disease development with limited tumor burden [22, 23]. Recently, Schatz [19] pointed out that Prostate cancer has several unique features that make it quite suitable for immunotherapeutic approaches. The timing and sequencing of various therapies for prostate cancer require special consideration. Thus, implementing immunotherapeutic approaches earlier in the prostate cancer disease spectrum would seem to be most appropriate.

We finally screened three genes related to prostate cancer recurrence: NDUFA13, UQCR11, USP34. Upon reviewing the relevant literature, we learned that gene NDUFA13(NADH: ubiquinone oxidoreductase subunit A13) encodes a subunit of the mitochondrial membrane respiratory chain NADH dehydrogenase (Complex I), which functions in the transfer of electrons from NADH to the respiratory chain. It has been reported that down-regulated NDUFA13 rendered tumor cells more resistant to apoptosis [24]. NDUFA13 inhibits cell growth of prostate cancer by regulating the expression of miR-423-5p [25]. These studies highlight the key role of NDUFA13 in tumor progression. The gene UQCR11 is differentially expressed in both lung adenocarcinoma [26] and breast cancer [27], but its specific significance in tumors has not been analyzed. Also, USP34 as a new player involved in the fine-tuning of NF-κB upon TCR stimulation [28]. The ability of the immune system to fight tumors was first discovered by Dr. William Coley in the nineteenth century. He used Coley’s toxins to trigger an immune response and treat patients with various types of inoperable carcinomas [29]. Although there is no literature on the specific relationship between prostate cancer and immune cells, Th2 cells and Tcm, there are more and more studies on the immune system and tumors. However, many studies have shown that the immune system plays an important role in malignant tumors such as breast cancer, ovarian cancer, prostate cancer, liver cancer and gastrointestinal cancer [30–32].

According to the results of this study, three key genes may affect the recurrence of prostate cancer after radical resection by affecting the infiltration of immune cells. Among them, the high expression of the gene USP34 may activate immune cells Th2 cells and Tcm to recognize tumors, thereby delaying recurrence and improving prognosis. Moreover, the high expression of the gene NDUFA13 and UQCR11 may inhibit the body’s immune system from recognizing tumors, and the probability of tumor recurrence will be significantly improved. We started with immune cell infiltration in prostate cancer tissues, combined with immune cell infiltration, to identify key genes for prostate cancer recurrence and immune cells. Validation showed that the key genes screened were significantly associated with the prognosis of prostate cancer. Further more, compared to the PSA and Gleason scores, model of two immune infiltrating cells and three key genes shows better prediction accuracy .

Finally, a nomogram was made based on two immune infiltrating cells and three key genes to establish a more accurate prediction model for prostate cancer recurrence. It was shown to provide a better accuracy for prostate cancer recurrence prediction after radical prostatectomy. Unfortunately, two immune cells infiltration and three key genes expression are not routine detection in clinical, but with the development of study in detection kit, the prediction model may help select patients at high risk for recurrence after radical prostatectomy.

These findings provide a new direction for prostate cancer treatment. Previous study showed that Th2 cells were significantly increased by Low-dose lymphocyte immunotherapy in patients of unexplained recurrent miscarriage [33], and PD-1/ PD-L1 blockade, one of the factors of cancer immune escape, has already been applied to clinical cancer therapy [34]. So, further we expect that Low-dose lymphocyte immunotherapy might be an effective therapy for prostate cancer, and even NDUFA13, UQCR11, and USP34 might be immunocheckpoints like PD-1/ PD-L1, they may play its role through Th2 and Tcm cells. Although this study did not provide insight into the specific mechanisms by which key genes pass through immune cells in prostate cancer recurrence, it points the way forward for our next study.

## Conclusions

This study found that immune cells Th2 and Tcm cells are associated with prostate cancer recurrence after radical prostatectomy, and they are independent protective factors. Infiltration of immune cells is linked to gene expression profiles by WGCNAand Lasso, discovering NDUFA13, UQCR11, and USP34 are prognostic biomarkers and associated with immune infiltration. Based on the study, we constructed a prediction model and plotted a nomogram, and this prediction model has better sensitivity and specificity for prostate cancer recurrence prediction.

## Data Availability

The data of this study are from GEO and TCGA database,

## Abbreviations

DCA: decision curve analysis
GEO: Gene Expression Omnibus
LASSO: Least absolute shrinkage and selection operator
PCa: Prostate cancer
ROC: receiver operating characteristic
ssGSEA: Single-sample gene set enrichment analysis
TCGA: The Cancer Genome Atlas
Tcm: central memory T cell
Th2: type 2 T helper
WGCNA: weighted gene co-expression network analysis
